# TEAMwork: Testing Emotional Attunement and Mutuality During Parent-Adolescent fMRI

**DOI:** 10.3389/fnhum.2020.00024

**Published:** 2020-02-07

**Authors:** Kara L. Kerr, Kelly T. Cosgrove, Erin L. Ratliff, Kaiping Burrows, Masaya Misaki, Andrew J. Moore, Danielle C. DeVille, Jennifer S. Silk, Susan F. Tapert, Jerzy Bodurka, W. Kyle Simmons, Amanda Sheffield Morris

**Affiliations:** ^1^Department of Human Development and Family Science, Oklahoma State University–Tulsa, Tulsa, OK, United States; ^2^Laureate Institute for Brain Research, Tulsa, OK, United States; ^3^Department of Psychology, The University of Tulsa, Tulsa, OK, United States; ^4^Department of Psychology, University of Pittsburgh, Pittsburgh, PA, United States; ^5^Department of Psychiatry, University of California, San Diego, La Jolla, CA, United States; ^6^Stephenson School of Biomedical Engineering, The University of Oklahoma, Norman, OK, United States; ^7^Janssen Research & Development, LLC, Johnson & Johnson, Inc., La Jolla, CA, United States

**Keywords:** fMRI, ventromedial prefrontal cortex, adolescence, parenting, emotion regulation

## Abstract

The parent-child relationship and family context influence the development of emotion regulation (ER) brain circuitry and related skills in children and adolescents. Although both parents’ and children’s ER neurocircuitry simultaneously affect how they interact with one another, neuroimaging studies of parent-child relationships typically include only one member of the dyad in brain imaging procedures. The current study examined brain activation related to parenting and ER in parent-adolescent dyads during concurrent fMRI scanning with a novel task – the Testing Emotional Attunement and Mutuality (TEAM) task. The TEAM task includes feedback trials indicating the other dyad member made an error, resulting in a monetary loss for both participants. Results indicate that positive parenting practices as reported by the adolescent were positively correlated with parents’ hemodynamic activation of the ventromedial prefrontal cortex, a region related to empathy, during these error trials. Additionally, during feedback conditions both parents and adolescents exhibited fMRI activation in ER-related regions, including the dorsolateral prefrontal cortex, anterior insula, fusiform gyrus, thalamus, caudate, precuneus, and superior parietal lobule. Adolescents had higher left amygdala activation than parents during the feedback condition. These findings demonstrate the utility of dyadic fMRI scanning for investigating relational processes, particularly in the parent-child relationship.

## Introduction

Emotion regulation (ER) involves emotional processes and influences on the expression and experience of emotions (Gross, 1998). This is done in service of a goal, whether this is a behavioral goal or simply the goal of feeling less (or more) intense emotions (Gross, 2015). Difficulties with ER are common in many psychiatric disorders, including depression (Joormann and Stanton, 2016), anxiety (Cisler et al., 2010), eating disorders (Oldershaw et al., 2015; Dingemans et al., 2017), borderline personality disorder (Gratz et al., 2016), oppositional defiant disorder (Cavanagh et al., 2017), and substance use disorders (Wilcox et al., 2016). Symptoms of many of these disorders often begin in adolescence. For example, rates of major depressive disorder sharply increase between the ages of 15 and 18 (Hankin et al., 1998), and difficulties in ER have been linked with adolescent depression (Silk et al., 2003; Fowler et al., 2017). Thus, a detailed understanding of the neurobiological processes involved in ER during adolescence is critical for the development of prevention and treatment for both internalizing and externalizing disorders.

The parent-child relationship is perhaps the most important context for ER development, and parenting practices likely affect the structure and function of ER neurocircuitry in children and adolescents. For example, harsh corporal punishment in childhood is related to reduced gray matter volumes in the medial prefrontal cortex, dorsolateral prefrontal cortex (dlPFC), and anterior cingulate cortex (ACC) in adulthood (Tomoda et al., 2009). Parenting also predicts the structural development of the amygdala and regions in the frontal cortex during adolescence, particularly in males (Whittle et al., 2014, 2016, 2017). Maternal warmth is related to lower fear-related amygdala hemodynamic activation in adolescents (Romund et al., 2016), indicating supportive parenting may lessen emotional reactivity. In contrast, high levels of parental psychological control, reflecting overprotection and intrusion into the child’s emotional experiences, are associated with attenuated anterior insula activity in adolescents in an emotion categorization task and with less accurate responding during the task (Marusak et al., 2018). These findings provide evidence that parental psychological control may constrain the development of ER skills in children, as the anterior insula can be considered an ER hub (Morawetz et al., 2017a). Finally, parent-child synchrony, an important concept with both behavioral and biological bases (Feldman, 2007), has recently been supported by resting-state fMRI findings (Lee et al., 2017). In this study, parents and adolescents with similar functional connectivity between resting-state networks also had similar daily emotional synchrony, and emotional synchrony was associated with higher levels of emotional competence in adolescents. Together, these studies provide a basis for understanding the importance of the parent-adolescent relationship in the development of brain structure and function underlying ER.

One important facet in the parent-child relationship is the emotional response when one person makes an error that affects the dyad (i.e., dyadic error processing). How parents respond when a child makes mistakes, whether harshly or constructively, may significantly impact the child’s socioemotional development as well as the parent-child relationship. Likewise, when the parent is at fault, the child’s response is likely reflective of the relationship, particularly as the child reaches adolescence and has greater agency. While there is little research on response to errors made by others, neuroimaging studies on individual error processing (i.e., one’s response to one’s own mistake) reveal that these tasks elicit activation in many of the same brain regions involved in ER, such as anterior insula and medial prefrontal cortex (Menon et al., 2001; Klein et al., 2007; Taylor et al., 2007; McCormick and Telzer, 2013). The realization that one has committed an error typically results in negative emotions that may need to be regulated in order to continue with task performance. Thus, error processing can involve implicit ER (Gyurak et al., 2011). Dyadic error processing constitutes a different phenomenon than that studied in traditional error processing research, as the construct here is how one responds when one’s partner, rather than oneself, commits the error. While few neuroscientific studies have examined dyadic error processing, one study reported that an observer’s relational closeness to the person committing an error modulated the observer’s neurophysiological responses to the error (Kang et al., 2010). This highlights the importance of the relational context in the study of interactive processes, such as dyadic error processing.

To our knowledge, no fMRI studies have yet simultaneously examined both parents and adolescents in real-time; however, a few have scanned adolescents while hearing recorded comments from their mothers (Lee et al., 2015; Aupperle et al., 2016). In a sample of healthy adolescents, Lee et al. (2015) found that listening to maternal criticism resulted in decreased activation in cognitive and social control networks and increased activation in subcortical-limbic regions associated with emotional reactivity. The authors of the study concluded that adolescents may not be effectively employing regulatory networks to modulate their emotional responding. In a similar study with a high-risk sample of adolescent girls, Aupperle et al. (2016) found that the right amygdala response to maternal criticism was positively correlated with symptoms of anxiety and depression, further underscoring the importance of studying the parent-adolescent relationship as a context for emotional development.

Neuroimaging studies have thus provided initial findings regarding the effects of parenting practices on the neurobiology underlying adolescent ER development (Kerr et al., 2019). These findings lend support to decades of observational and behavioral data on the importance of the parent-child relationship and emotional development. The neurobiology underlying the social processes that constitute this relationship, however, remain unexplored. Past studies have largely focused on the adolescents’ brain responses in relation to reported parenting practices (e.g., Romund et al., 2016; Marusak et al., 2018) or relationships between adolescents’ and parents’ brain responses when completing the same non-social task or resting-state scan independently (e.g., Colich et al., 2017; Lee et al., 2017). While we focus here on the parent-child relationship, a similar paucity of research using ecologically valid social tasks exists across other domains as well. Indeed, neuroimaging has lagged behind other methodologies, particularly observational studies, in its ability to examine inter-individual processes. Novel experimental paradigms, and particularly fMRI tasks that reflect social processes within important relationships, are needed to address these knowledge gaps.

We therefore developed an fMRI task designed to probe emotional reactivity and regulation in a dyadic context – the Testing Emotional Attunement and Mutuality (TEAM) task. This task builds on past studies that have utilized error processing paradigms in the study of ER (e.g., Lewis et al., 2007; Ichikawa et al., 2011; Levsen and Bartholow, 2018) by specifically examining dyadic error processing. Parent-adolescent dyads completed the TEAM task while simultaneously undergoing fMRI scanning. This task was developed to examine brain activation in both parents and adolescents when the other member of the parent-adolescent dyad makes a costly error. It thus allows us to probe emotion reactivity and regulation in response to being “let down” by the other person within the context of a relationship and processing an error made by a family member. The costly errors result in a monetary loss for the dyad, which evokes negative affect in participants, particularly because they themselves responded accurately (Angus and Harmon-Jones, 2019). In order to continue performing the task on the next trial, participants must regulate their emotional response to their partner’s error (implicit ER). As part of the study, adolescents were also asked to report on their parents’ positive parenting practices, thus allowing us to determine how a parent’s brain response to their child’s costly error may be correlated with parenting behavior in daily life. We had three main hypotheses: (1) positive parenting practices would be positively correlated with parents’ activation of ER-related brain regions, indicating a regulatory response to their child’s error, (2) both parents and adolescents would exhibit activation in brain regions underlying emotional reactivity and regulation and error processing in response to the other dyad member making a costly error, and (3) as compared to parents, adolescents would have greater activation in regions related to emotional reactivity (e.g., amygdala) and less activation in regions related to ER (e.g., dlPFC).

## Materials and Methods

### Participants

This study was conducted at the Laureate Institute for Brain Research (LIBR) with research protocol (IRB # 2017011) approved by the Oklahoma State University Center for Health Sciences Institutional Review Board (IRB). Adolescents aged 14–16 years participated in the current study with one of their biological parents. Participants were recruited from flyers posted in the community and electronically distributed through local schools. Adult participants provided written informed consent for their own and their adolescent’s participation, and adolescent participants provided written informed assent. Consent was obtained in accordance with the Declaration of Helsinki. All participants received financial compensation for participation.

Exclusion criteria for both adolescents and parents included history of major medical or neurological disorders, left-hand dominance [assessed by the Edinburgh Handedness Inventory (Oldfield, 1971)], current pregnancy, psychotropic medication use (other than stimulant medications) within the past 3 weeks (6 weeks for fluoxetine), stimulant medication use within 36 h prior to the scan, or meeting general MRI exclusion criteria (e.g., ferrous metal implants). Participants were also excluded if they met criteria for any current psychiatric diagnosis as assessed by the Mini International Neuropsychiatric Interview [MINI 7.0 (Sheehan et al., 1997); MINI KID 7.0 (Sheehan et al., 2010)], and adolescents were additionally excluded for any history of psychiatric disorder, with the exception of attention-deficit/hyperactivity disorder. These criteria were assessed by a phone screen interview with the parent and an initial screening visit. Clinical interviews were administered by trained personnel supervised by a licensed clinical psychologist. Forty parent-adolescent dyads met initial inclusion criteria and returned for the scanning session. This sample size was selected based on typical samples in related studies.

After data acquisition, participants were excluded for excessive motion while in the scanner (e.g., average motion across all runs >0.15, based on AFNI’s “enorm” value reflecting the Euclidean-normalized motion derivatives), other issues resulting in poor data quality (e.g., signal artifact from orthodontic retainers), or technical difficulties (e.g., problems with the response box). One participant was excluded for indicating during debriefing that during the task they began suspecting it was pre-programmed. Data quality criteria were applied to individual participants rather than dyads; thus, participants were included in analyses if they met all criteria, regardless of the inclusion of the other dyad member. This resulted in a final sample of 25 parents (age range: 30–53 years) and 27 adolescents (age range: 14–16 years) from an original sample of 40 dyads. Please see Table 1 for demographic information.

**TABLE 1:** Sample demographics.

	Parents (*n* = 25)	Adolescents (*n* = 27)
Female	23	15
Age (years; *M* [*SD*])	42.92 (5.73)	14.89 (0.89)
**Parent education**		
High school graduate/GED	3	–
Some college/trade school	4	–
College graduate	13	–
Graduate degree	5	–
**Race**		
African American	2	3
Caucasian	22	22
Multiple races	1	2
**Ethnicity**		
Hispanic or Latinx	3	2
Not Hispanic or Latinx	22	25

### Procedures and Measures

Participants first completed a screening visit, during which they completed the MINI or MINI KID, MRI screening, demographics survey, and measures of behavior and mental health. Adolescents completed the 42-item Alabama Parenting Questionnaire (Frick, 1991), which includes subscales assessing a parent’s involvement, positive parenting practices, monitoring/supervision, inconsistent discipline, and corporal punishment. Adolescents responded to each item on a five-point Likert scale. The positive parenting subscale (APQ-Pos) was selected as the primary parenting measure for the current study (Cronbach’s α for the current sample = 0.76), as positive parenting is associated with mental health and self-efficacy in adolescents (Tabak and Zawadzka, 2017). Examples of items from this scale include “Your parents tell you that they like it when you help out around the house” and “Your parents tell you that you are doing a good job.”

Participants meeting inclusion criteria based on the initial phone screen and in-person screening visit returned for a second session, during which they completed the fMRI scan. Prior to scanning, participants were screened for current alcohol and drug use, and female participants were screened for pregnancy. Participants were provided instructions for the tasks they were to complete in the scanner and were given the opportunity to briefly practice these tasks before the scan on a laptop computer. Participants also engaged in a motion detection paradigm in a mock scanner to practice lying still and acclimate to the scanning environment.

### Task

**FIGURE 1:**
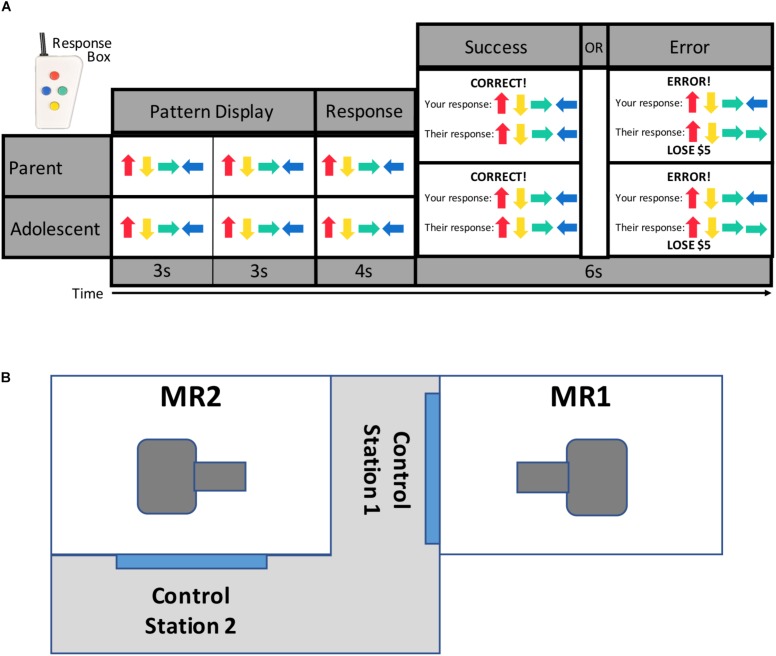
TEAM task design and scanner configuration. Parents and adolescents were scanned simultaneously while each performing the TEAM task **(A)**. The TEAM task consists of trials during which a pattern of four arrows is displayed twice to the participant, and the participant then enters the pattern from memory using a handheld response box. Participants are then provided feedback about both their own and their partner’s performance. Unbeknownst to the participant, however, the feedback for their partner’s performance has been pre-programmed to either show a correct (14 trials per run) or incorrect (three trials per run) response. Scanners are located in close physical proximity separated by a shared control room **(B)**.

The newly developed TEAM task (Figure 1) was presented to each dyad as a cooperative game. Participants completed the task while simultaneously undergoing fMRI scanning. The TEAM task is an event-related design and consists of 17 trials during which participants first see a pattern of colored arrows presented sequentially on the screen for 3 s, twice in a row (totaling 6 s). They are then given 4 s to reconstruct the sequence by pressing colored buttons on a response box (shown in Figure 1). At the end of the 4-s response window, participants see a message with feedback regarding both dyad members’ performance on that trial. Prior to the scan, participants are told that if one or both members of the parent-adolescent dyad respond incorrectly to a trial, they will lose $5 from a starting amount of $50.

Participants are told that they are completing the task cooperatively with the other dyad member (their parent or child), but in reality, the feedback for their partner was pre-programmed with three trials per scanning run revealing that their partner made an error. All other trials show that their partner responded correctly. The participant always receives accurate feedback regarding his or her own performance. The task was pre-programmed to ensure there would be trials where the partner was incorrect but the participant was correct, as the task was simple enough that most participants made few or no errors (average error rate across both runs = 1.81 trials). The task was therefore designed in such a way that the participant was likely to perceive the task as fairly easy, thus enhancing the salience of their partner’s errors.

The TEAM task additionally includes “s-detection” trials (data not presented here) during which participants see a random 20-letter string for 6 s and are asked to press a button if the string includes the letter “s.” These trials were developed as an active baseline condition. Participants do not receive any feedback about their performance on these trials, and no money is at stake. Participants completed two runs of the TEAM task, each lasting 7 min and 50 s. Each run included 17 pattern trials (3 of which showed a partner error) and 14 s-detection trials. Runs also included inter-stimulus intervals with a white screen and black fixation mark ranging from 2 to 10 s. Participants were debriefed following completion of the TEAM task and informed that they would receive the full $50.

E-Prime 2 software^1^ was used for stimulus presentation and behavioral data collection. Stimuli were presented via front projection. Stimulus presentation for each run was triggered and synched to the scanner by TTL pulse.

### MRI Data Acquisition

Two identical (e.g., hardware and software configuration) General Electric Discovery MR750 whole-body 3 Tesla MRI scanners in close physical proximity were used to acquire the functional and structural brain images with whole-brain coverage (Figure 1B). Because the task was pre-programmed, the scans were run independently (though at the same time) and not linked. For MR signal reception, system-provided receive-only 8-element surface coil head coils were used. Blood-oxygenation level-dependent (BOLD) fMRI scans were obtained with a single-shot gradient-recalled EPI sequence with sensitivity encoding (SENSE). The following EPI parameters were used: FOV/slice/gap = 240/2.9/0 mm, 41 axial slices per volume, acquisition matrix = 96 × 96, repetition/echo time (TR/TE) = 2000/25 ms, SENSE acceleration factor *R* = 2 in the phase encoding (anterior–posterior) direction, flip angle = 78°, sampling bandwidth = 250 kHz, number of volumes = 235, scan time = 7 min and 50 s. EPI images were reconstructed into a 128 × 128 matrix, with an fMRI voxel volume of 1.875 mm × 1.875 mm × 2.9 mm. Scanners are equipped with real-time motion monitoring.

A T1-weighted MRI scan with magnetization-prepared rapid gradient echo (MPRAGE) sequence with SENSE was used for structural MRI and anatomical reference for the fMRI analyses. The following parameters were used for MPRAGE sequence: FOV/slice = 240/0.9 mm, 180 axial slices per volume, image matrix = 256 × 256, voxel volume = 0.94 × 0.94 × 0.9 mm^3^, TR/TE = 5/2.012 ms, SENSE acceleration factor *R* = 2, flip angle = 8°, inversion/delay time (TI/TD) = 725/1400 ms, sampling bandwidth = 31.25 kHz, scan time = 6 min and 13 s.

### Imaging Data Analysis

All analyses were performed using AFNI^2^. Preprocessing steps were achieved utilizing afni_proc.py. The anatomical scan was aligned to the first volume of the EPI data, followed by spatial normalization to the stereotaxic array of Talairach and Tournoux (1988). The first four fMRI volumes were excluded from analysis to allow the signal to reach steady state. Spatial normalization and motion correction were implemented in a single image transformation. The EPI data were resampled to a 1.75 × 1.75 × 1.75 mm grid and spatially smoothed with a 6 mm full-width at half-maximum Gaussian kernel. Each voxel’s signal timecourse was normalized to reflect percent signal change from the voxel’s mean signal. Individual time points were censored if their Euclidean-normalized motion derivative exceeded 0.3 or if the fraction of voxels considered outliers based on 3dToutcount’s “automask” function exceeded 0.05.

### Statistical Analyses

**FIGURE 2:**
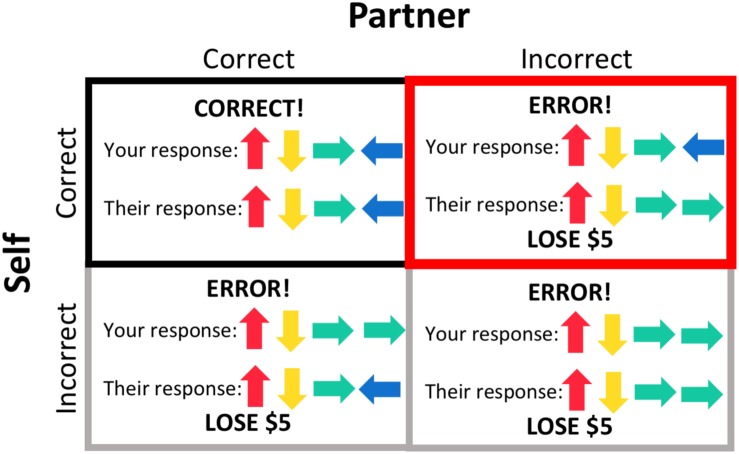
Feedback conditions. The outcome of each trial resulted in one of four feedback conditions, based on the participant’s response and their partner’s response, the latter of which was pre-programmed. Trials during which the participant gave an incorrect response **(gray borders)** were included as regressors in subject-level processing but not included in group-level analyses. Feedback that both the participant and their partner responded correctly **(black border)** was used as a baseline for the condition of interest, the “costly error” condition, when the participant responded correctly but their partner did not, resulting in a loss of $5 **(red border)**.

A general linear regression model was used to analyze data at the single participant level. Regressors of interest included the s-detection trials, pattern display, response period, and four different feedback conditions (Figure 2) – both the participant and their partner responded correctly (“both correct”); the participant responded correctly but their partner responded incorrectly, resulting in a loss of $5 (“costly error” condition); the participant responded incorrectly, resulting in a loss of $5 (but their partner responded correctly); and both the participant and their partner responded incorrectly, resulting in a loss of $5. These regressors were individualized, and thus some participants did not have regressors for one or both of the conditions where the participant responded incorrectly (if they always entered the correct response). Task regressors were constructed by convolving a series of gamma-variate functions beginning at the onset of the condition. The regression model also included regressors of non-interest that accounted for each run’s signal mean, linear, quadratic, and cubic signal trends and six motion parameters (three translations and three rotations) computed during the image registration preprocessing.

We were primarily interested in participants’ brain activation in response to feedback that their partner made an error (but they themselves responded correctly), resulting in a loss of $5 for the dyad, as opposed to receiving feedback that both dyad members responded correctly. We therefore used the “both correct” feedback condition as a comparison condition for this analysis. AFNI’s 3dttest++ program was used to evaluate task effects within each group (parents and adolescents) and to conduct the group comparison. Beta coefficients entered into each analysis represented the contrast between the “costly error” and “both correct” conditions. We additionally conducted group comparisons within the adolescent sample to determine if there were any effects of self-reported participant gender. We did not have adequate power to test the effects of participant gender in the parent sample.

In order to examine relationships between parent and adolescent brain activation, we conducted an additional analysis utilizing a subsample of 17 dyads with data available for both the parent and the adolescent. The AFNI program 3dTcorrelate was used to identify regions that were significantly correlated (using the Spearman method) between parents and their adolescent children. Beta coefficients for the “costly error” trials with the “both correct” trials as a baseline were entered into this analysis. Additionally, it should be noted that although constructs such as parent-adolescent synchrony and cross-brain connectivity will be important in furthering our understanding of the parent-adolescent relationship, we chose to focus on task-based analyses in this study for two primary reasons. First, while parents and adolescents were told they were receiving the same feedback as their partner, this was actually not the case (i.e., each thought that the other person made the error). Parent-adolescent synchrony and cross-brain connectivity have the underlying presumption that parent-adolescent dyads have a similar and/or interactive response to shared stimuli. Second, analyses such as inter-subject correlations rely on comparing participants’ brain activity across time. As an event-based paradigm, the salient events in the TEAM task are the “costly error” feedback trials. The rest of the time, participants are essentially performing a working memory task that is largely unrelated to dyadic emotion reactivity and regulation. Nevertheless, strengths of our approach include targeting salient, emotionally evocative events in a dyadic context, as well as the ability to examine both parent and adolescent neurocircuitry in a cooperative task.

In order to determine if parents’ brain responses to their child’s costly error were related to behavioral differences in parenting, we used AFNI’s 3dTcorr1D to identify brain areas in the parent sample where activation in response to the costly error (with “both correct” as a baseline) was correlated with APQ-Pos scores. We initially conducted this analysis using traditional Pearson correlations; however, when we examined the resulting scatterplots, it was apparent that this method resulted in a few false positives due to outliers. These outliers were typically different individuals in the different brain regions exhibiting significant effects. We therefore chose to instead conduct Spearman correlations to ensure any effects were robust to the presence of outliers. We additionally conducted an exploratory analysis using the same method to examine possible relationships between APQ-Pos scores and adolescents’ brain activation in response to the costly error.

A whole-brain mask was created using AFNI’s 3dmask_tool. The whole-brain mask was defined as all voxels with at least 70% overlap of all subjects’ individual brain masks based on their EPI data, as generated by 3dAutomask. In addition to the whole-brain mask, we also performed cluster-size corrections within anatomically defined regions of interest (ROIs) selected *a priori* due to their known involvement in emotion processing and regulation – ventromedial prefrontal cortex (vmPFC), dlPFC, amygdala, and insula. (Please see below for descriptions of how these regions were defined.) To control and limit false-positive detection, for the whole brain mask and each ROI we used a voxelwise threshold of *p* < 0.005 with a cluster-size threshold of *p* < 0.05, based on AFNI’s 3dClustSim procedure utilizing the “‘-acf” option, which uses spherical autocorrelation parameters from 3dFWHMx with non-Gaussian filtering (Cox et al., 2017).

### Anatomical ROI Definitions

All ROIs were anatomically defined *a priori*. Because there is not a widely accepted standard anatomical definition for the vmPFC, the vmPFC ROI mask was drawn from a collection of masks (Clausen et al., 2017) that were created using a multi-step, data-driven approach. High-resolution T1-weighted images (172, 1 mm thick slices) from a sample of 43 healthy adults (none of which were in the present study) were separated into gray and white matter and normalized to Talairach space using SPM5 (Statistical Parametric Mapping software)^3^. The gray matter maps were combined to make a voxel-wise gray matter probability map, to which Talairach stereotactic definitions were applied, resulting in 76 Talairach-defined brain regions. Gray matter probabilities (ranging from 5–50% in increments of 5) and dilation (from 8 mm^3^ to 64 mm^3^) clip level (ranging from 5–50% in increments of 5) were then used to created maps of brain regions. The overlap of each map with the stereotactic atlas definition was next used to calculate the sensitivity and specificity of each map with the stereotactic atlas definition. These values were then plotted on a receiver-operator curve, and the map with maximum sensitivity and specificity for each region was selected as the optimal mask. The vmPFC ROI masks extends to the edge of the brain anteriorly and ventrally. Its dorsal edge extends up to but not including the ACC. Laterally, it extends to *x* = ±18. For further details regarding this mask, as well as a visual representation, please see Clausen et al., 2017.

Separate masks for each hemisphere were used for the remaining ROIs. For the amygdala and insula ROIs, we used pre-rendered ROI masks available in AFNI. The pre-rendered masks are based on probability maps for various cortical areas (Desikan et al., 2006) in conjunction with the parcellation of cortical and subcortical structures generated by the FreeSurfer program based on the Talairach N27 atlas brain in AFNI. The amygdala ROI mask was edited to extend to the posterior edge of the amygdala as defined in the Mai atlas (Mai et al., 2007). A pre-rendered ROI mask from AFNI was also used for the dlPFC. This mask was defined as Brodmann area 45 based on a maximum probability map of the Talairach N27 atlas brain.

## Results

### Parents’ Brain Responses and Positive Parenting

**FIGURE 3:**
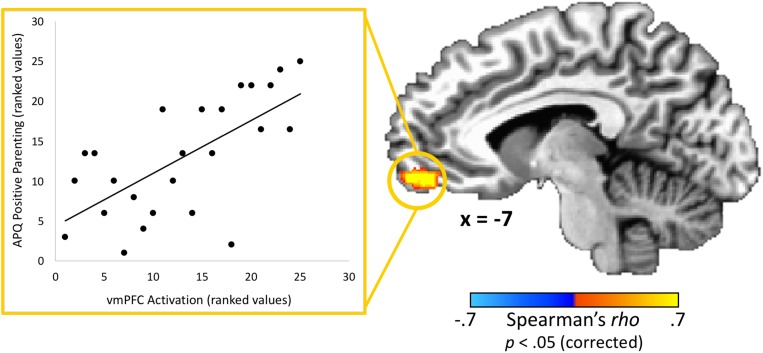
Relationship between adolescents’ ratings of their parents’ positive parenting and parents’ brain response to their adolescent child’s costly error. A region-of-interest analysis revealed that the vmPFC exhibited a significant positive correlation between positive parenting practices (as reported by the adolescent) and parents’ brain response when their child made a costly error. Higher vmPFC activation was associated with positive parenting practices. Scatterplot data represent mean activation in the identified region (ranked within-group) and are provided for visualization purposes only. Coordinates are in Talairach space.

With regard to associations with parenting practices, one brain region – the vmPFC – had a statistically significant correlation (peak *rho* = 0.75; *x* = 1, *y* = 59, *z* = −5; volume = 986 mm^3^) between parents’ activation and positive parenting practices as measured by the APQ-Pos scale (completed by the adolescent) after corrections for multiple comparisons (Figure 3). This indicates that parents whom adolescents rated as exhibiting the most positive parenting practices in daily life also had the highest vmPFC activity when receiving feedback that their adolescent child made a costly error. Conversely, those parents who failed to activate this region in response to learning that an adolescent child had made a costly error received the poorest parenting ratings from adolescents. Because the “both correct” trials were utilized as a baseline for the “costly error” trials, we performed a follow-up analysis to ensure results were not driven by effects from this condition. We calculated the average beta values for each condition within the vmPFC cluster identified as exhibiting a significant correlation with positive parenting. Results supported that the significant correlation was driven by the costly error trials (Spearman’s *rho* = 0.53, *p* < 0.01) rather than the “both correct” trials (Spearman’s *rho* = −0.13, *p* = 0.53). Of note, although it did not pass corrections for multiple comparisons, we also identified a region in the right posterior insula showing an association (peak *rho* = 0.71; *x* = 39, *y* = −8, *z* = −5; volume = 139 mm^3^) with positive parenting at a corrected *p* = 0.06. No significant correlations were found between APQ-Pos scores and adolescents’ brain activation in response to the costly error trials.

### Task Effects for Parents and Adolescents

**FIGURE 4:**
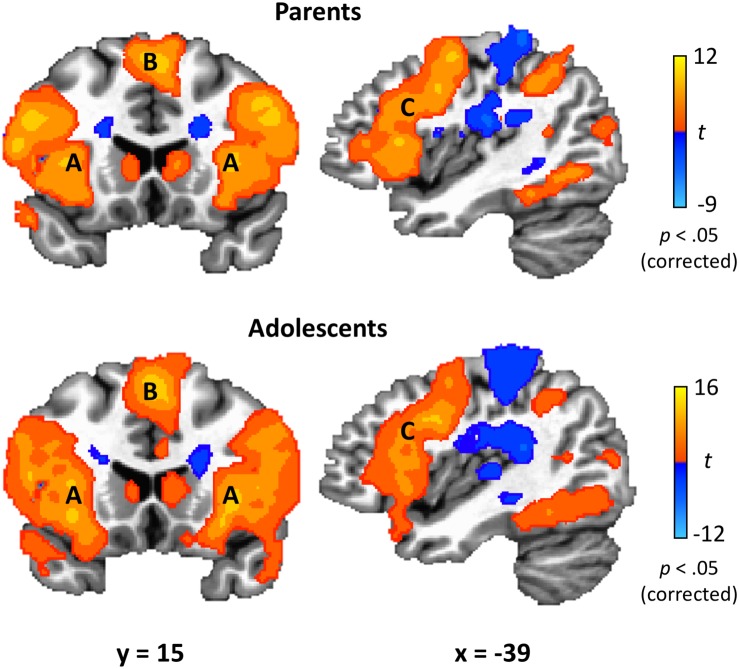
TEAM task results. In response to their partner’s costly error, both parents and adolescents exhibited robust activation in brain regions related to affective circuitry and emotion regulation, including anterior insula **(A)**, medial superior frontal gyrus **(B)**, and dorsolateral prefrontal cortex **(C)**. Coordinates are in Talairach space.

Parents’ and adolescents’ results for the TEAM task are displayed in Figure 4. In response to feedback that their partner made a costly error, both parents and adolescents exhibited heightened activity bilaterally in the dlPFC, anterior insula, fusiform gyrus, thalamus, caudate, precuneus, and superior parietal lobule (Table 2). Both groups had decreased activity relative to the “both correct” condition in areas bilaterally including the postcentral gyrus, cuneus, and ACC. There was only one significant group difference between adolescents and parents in response to their partner making a costly error: adolescents exhibited greater activation in the left amygdala (peak *t* = −3.87; *x* = −17, *y* = −8, *z* = −7; volume = 225 mm^3^). There was also a significant gender difference in the vmPFC in the adolescent sample (peak *t* = 3.97; *x* = −1, *y* = 48, *z* = −10; volume = 263 mm^3^), with males showing greater activation than females.

**TABLE 2:** Within-group effects for “costly error” – “both correct” contrast.

	Talairach coordinates				
	
Region	*x*	*y*	*z*	Peak *t*	Volume (mm^3^)
**Parents**	−	−	−	–	–
Postcentral gyrus/R temporoparietal junction/R posterior insula	25	–41	60	–8.55	58278
Dorsomedial prefrontal cortex/R dorsolateral prefrontal cortex/R anterior insula	41	20	9	12.03	56509
L dorsolateral prefrontal cortex/L anterior insula	–55	18	14	9.48	40538
Thalamus/caudate/R pallidum/R parahippocampal gyrus/R fusiform gyrus	–4	–27	0	9.76	33882
R inferior/superior parietal lobule/R precuneus/R middle occipital gyrus	31	–59	42	10.33	19739
L inferior/superior parietal lobule/L precuneus/L middle occipital gyrus	–24	–66	39	8.31	16823
Cuneus/R lingual gyrus	20	–90	14	–7.14	15794
L fusiform gyrus/L middle temporal gyrus	–31	–50	–10	7.09	11089
Anterior cingulate	18	38	4	–6.13	11078
L parietal operculum/L dorsal mid-insula/L posterior cingulate	–39	–22	20	–5.67	10226
L dorsal anterior cingulate	–18	–17	30	–5.01	2401
R dorsal mid-insula	32	4	16	–4.92	311
L amygdala	–22	–5	–10	4.71	118
**Adolescents**	−	−	−	–	–
L dorsolateral prefrontal cortex/L anterior insula/thalamus/caudate/parahippocampal gyrus/amygdala/L superior temporal gyrus/L fusiform gyrus/cerebellum	–29	20	2	13.81	94523
Postcentral gyrus	–11	–38	58	–11.61	57088
R dorsolateral prefrontal cortex/R anterior insula	32	18	6	15.61	48031
R temporoparietal junction/R posterior insula/anterior cingulate/R putamen	59	–12	11	–8.07	43030
L temporoparietal junction/L posterior insula/L putamen	–34	–22	7	–7.58	25966
Dorsomedial prefrontal cortex	–1	17	49	12.96	24600
R inferior/superior parietal lobule/R precuneus/R middle occipital gyrus	41	–43	37	8.67	21228
L inferior/superior parietal lobule/L precuneus/L middle occipital gyrus	–24	–62	41	10.66	17348
Cuneus	15	–89	14	–9.67	14953
R fusiform gyrus/R middle temporal gyrus	34	–40	–17	7.16	5793
L middle temporal gyrus	–45	–29	0	6.28	4695
L superior frontal gyrus	–22	34	49	–5.66	3098
R lingual gyrus/cerebellum	20	–48	–19	–4.47	2455

*Regions are bilateral unless otherwise noted. R, right; L, left.*

In addition to the main contrast of interest, we additionally examined results for each condition (“costly error” and “both correct”) independently with a resting baseline, although fMRI effects with a resting baseline should be interpreted with caution (Stark and Squire, 2001). Results of these analyses, along with statistical maps for the “costly error” condition with “both correct” as a baseline (described above), can be found at NeuroVault.org (Gorgolewski et al., 2015). Data can be accessed at https://identifiers.org/neurovault.collection:6150.

### Inter-Brain Correlation

A Spearman correlation analysis was performed to examine any significant relationships between parents’ and adolescents’ brain activation in response to the costly error trials (with “both correct” trials as a baseline). A subsample of 17 dyads with fMRI data suitable for analysis was included. After corrections for multiple comparisons, only one region – left anterior insula – exhibited a significant correlation between parents and adolescents (peak Spearman’s *rho* = 0.79; *x* = −25, *y* = 25, *z* = 9; volume = 187 mm^3^).

## Discussion

This study demonstrates the utility of an ecologically valid task for studying parent-adolescent dyadic error processing during neuroimaging. Results revealed how parenting behaviors relate to brain activation in response to an adolescent’s error: positive parenting practices, as perceived by the adolescent, correlated with greater activation in the parents’ vmPFC, a key region involved in empathy and ER and emotion processing. Additionally, in both parents and adolescents, reactions to the other dyad member making a costly error resulted in activation in other key emotion processing regions, including dlPFC, anterior insula, fusiform gyrus, thalamus, caudate, and superior parietal lobule. Thus, at this stage of development, the neurobiology underlying one’s response to being “let down” by a family member seems to be similar for both parents and adolescents. These findings provide initial evidence of the utility of dyadic fMRI for studying socioemotional processes.

This dyadic neuroimaging paradigm allowed us to investigate how parents’ brain responses to their children’s errors might reflect positive parenting behaviors. Our analyses revealed that activation of the vmPFC was positively correlated with adolescent-reported positive parenting behaviors, such as offering praise and positive reinforcement. Past research has implicated the vmPFC in a myriad of different processes. Recently, Hiser and Koenigs (2018) reviewed the vmPFC’s various functions in emotion, decision-making, moral judgments, and social cognition. They noted that the vmPFC plays a critical role in social cognitive processes such as perspective-taking and emotion recognition through its interaction with other regions activated during the TEAM task, including the dorsomedial prefrontal cortex, amygdala, and precuneus. Notably, the vmPFC’s role in perspective-taking appears to be specific to affective components (Healey and Grossman, 2018). Individuals with vmPFC lesions perform poorly on affective perspective-taking tasks, even when compared to individuals with lesions in other prefrontal areas (Shamay-Tsoory et al., 2005, 2006; Shamay-Tsoory and Aharon-Peretz, 2007). Taken together with our findings, this suggests that positive parenting practices are particularly associated with an emotional understanding of the child’s experience.

The vmPFC’s role in socioemotional processes is also supported by the extensive literature on lesions to the vmPFC. A recent review of these studies (Schneider and Koenigs, 2017) provides additional insight into the various functions of the vmPFC, including its role in social cognition and empathy. The expression and recognition of emotions, which are fundamental to social cognition and functioning, are both impaired in individuals with vmPFC lesions (Damasio et al., 1990; Hornak et al., 1996; Heberlein et al., 2008; Tsuchida and Fellows, 2012; Monte et al., 2013; Vandekerckhove et al., 2014). Positive parenting is grounded in these emotion processes, as it requires both being able to recognize a child’s emotions and to effectively express one’s own emotions, with behaviors such as smiling and expressing pride in a child’s behavior. More recently, Beadle et al. (2018) provided experimental evidence for reduced empathy in a sample of individuals with damage to the vmPFC. In an empathy induction task, as compared to healthy individuals and those with damage to other brain regions, individuals with vmPFC damage gave less money to an individual (research confederate) who was suffering. Our findings therefore indicate that parents with more empathic brain responses to their child’s error, as evidenced by vmPFC activation, also engage in more positive parenting behaviors in daily life as reported by their child. Much of the past research on empathy and parenting has been related to the child’s development of empathy rather than parental empathy (e.g., Yoo et al., 2013; Van Lissa et al., 2015). The current study adds to this literature by highlighting the importance of parental empathy and its underlying neurobiology for positive parenting behaviors, which likely impact adolescent mental health (Tabak and Zawadzka, 2017).

Study findings also supported the hypothesis that our newly developed task would effectively probe activation of brain regions known to underlie ER. In response to their partner making a costly error, both parents and adolescents activated dlPFC, anterior insula, and the superior parietal lobule – three regions found in a meta-analysis of ER studies (Buhle et al., 2014). This meta-analysis specifically examined studies of cognitive reappraisal, where participants are asked to change their thoughts about an emotional stimulus. In contrast, in the current study, participants were not given specific instructions on how to respond to their partner’s mistake. Activation of these regions therefore indicates that participants may be engaging in implicit ER in order to recover from the negatively valanced event (i.e., losing $5 due to their partner’s error, disappointment due to their partner letting them down) and maintain goal orientation to perform on the next trial.

Further evidence for task-related ER is seen when looking more specifically at the individual brain regions that were activated. For example, anterior insula activity has been found to influence the function of other brain regions related to ER (Sridharan et al., 2008; Morawetz et al., 2017b), and adolescents’ regulation of anterior insula activity through neurofeedback drives subsequent activity in the broader ER network (Cohen Kadosh et al., 2016). Structurally, thinning of the dlPFC associated with maturation predicts ER skills in adolescent girls years later (Vijayakumar et al., 2014). Similarly, dlPFC activation is related to successful cognitive reappraisal of emotional stimuli in healthy adolescents but not in depressed adolescents (LeWinn et al., 2018). Together, these findings provide evidence that both parents and adolescents responded to feedback that their partner made an error with activation in ER-related regions.

In addition to regions implicated in ER, both parents and adolescents responded to their partner’s error with activation in brain regions related to perspective taking. The precuneus, which was activated in our task, underlies both cognitive and affective components of perspective-taking (Vollm et al., 2006; Sebastian et al., 2012; Schlaffke et al., 2015; Healey and Grossman, 2018). Our task also resulted in activation of regions related specifically to cognitive components of perspective-taking, namely dorsomedial prefrontal cortex and dlPFC (Healey and Grossman, 2018), as well as regions specifically related to affective components, such as the anterior insula (Kanske et al., 2015). Many past studies have implicated the anterior insula in empathy-related processes. Anterior insula activation during an emotion attribution task was found to be correlated with greater trait-like self-reported personal distress in response to the distress of others (Haas et al., 2015), suggesting a relationship with habitual empathic responding. Similarly, anterior insula activation when observing social exclusion was related to empathy and subsequent prosocial behavior (Masten et al., 2011). The correlation between parents’ and adolescents’ anterior insula activation provides initial evidence for parental influences on ER and empathy development on a neurobiological level. A past study of adolescents found that activation of the dorsomedial prefrontal cortex and vmPFC was associated with empathic accuracy (Kral et al., 2017), indicating that these regions are not simply involved in feeling empathic but are needed to correctly assess another person’s feelings. Yet another empathy-related region activated by the error condition was the caudate. While caudate activation has been associated generally with affective, empathy-related perspective-taking (Schlaffke et al., 2015), of particular relevance to the current study is the past finding that the caudate was activated when participants were asked to respond with ‘unconditional love’ to people shown in pictures (Beauregard et al., 2009).

Many of these brain regions have also been found in other studies of parenting. For example, fathers exhibit anterior insula activity in response to their infants’ cry (Mascaro et al., 2014). Another study found that in mothers, anterior insula activation to their infants’ cry was related to empathic behaviors (i.e., “mental state talk”) observed during interactions with their infant (Hipwell et al., 2015). Other regions activated by our task have also been found in studies of parental response to their infants, including the caudate, thalamus, and precuneus (Wan et al., 2014; Paul et al., 2019). Both precuneus and fusiform gyrus activation in response to infant cry increased following an attachment-based parenting intervention (Swain et al., 2017), indicating that these regions may be important for positive parent-child relationships. While there is comparatively little research on the neurobiology underlying the parenting of adolescents as compared to infants and young children, our findings point toward a common neural circuitry of parenting that extends across development from an infant’s cry to an adolescent’s mistake.

While most of our findings were predicted based on prior literature, an unexpected finding was a gender difference in vmPFC activation in response to the parents’ error, with adolescent males exhibiting greater vmPFC activation than females. Most of our adult participants were mothers, and this effect might therefore be due to an interaction with the same-versus opposite-sex parent. For example, one past study found that adolescent vmPFC activation during a delay discounting task in both males and females was related to the parenting practices of the same- but not the opposite-sex parent (Schneider et al., 2014). Future studies with a larger sample and more fathers participating will be necessary to disentangle these effects.

Our findings also revealed that adolescents exhibited greater activation in the left amygdala during the feedback condition as compared to parents. Past research on ER in adolescents has found decreasing amygdala reactivity across development in late adolescence despite having similar levels of prefrontal activation as compared to young adults (Stephanou et al., 2016). This corresponds with our findings, as the adolescents in our sample did not differ from adults in activation of regions such as the dlPFC. Thus, this difference between adolescents and parents likely reflects a developmental difference in amygdala reactivity.

This study presents a new paradigm that can be applied across different dyadic contexts (e.g., sibling pairs, romantic partners, coworkers). The study, however, has a few limitations. The sample size was relatively small for the group comparisons of parents (*n* = 25) and adolescents (*n* = 27) and males and females within the adolescent sample. However, overall patterns of activation from the task were quite similar between groups, demonstrating that the task results in brain activation in expected regions in an overall sample of 52 adolescent and adult participants. Another limitation is the small number of error trials (three per run) included in the task. Nevertheless, the error feedback was sufficiently provocative to render robust BOLD responses with even a small number of trials. Importantly, only a small number of error trials are possible in the design, as a large number of partner errors would likely reduce the believability of the task. Finally, the ecological validity of the task necessitated that the specific mental processes involved could not be isolated from the socioemotional context of the dyadic paradigm. Based on past research on the parent-child relationship and our understanding of the task, we have largely framed our results in the context of ER. However, other processes, including dyadic error processing, are also simultaneously occurring, and it is difficult to extricate these effects from the current task design. Participants were not given any instructions on how to respond when they or their partner made an error, and thus the evoked brain activation represents how they were likely to respond in a similar situation outside the scanner.

Our study presents a unique and ecologically valid paradigm with the potential for broad applications. We utilized fMRI to simultaneously examine the brain activation of both individuals in a dyadic context. This is a novel aspect of our study, as most previous research has focused on either the parent or the child, but rarely both. The ability to scan two individuals simultaneously uniquely allows for the examination of both individuals in a dyadic context rather than scanning each person individually and comparing their brain activation. However, a strength of this paradigm is also its applicability outside of the dyadic scanning context. Researchers who are primarily interested in one member of the dyad can scan that individual, who would be told that the other member is completing the task outside of the scanner. Additionally, our results indicate that this paradigm is useful in probing processes related to ER as well as empathy and perspective taking. These processes are evoked implicitly and naturalistically, as participants are not given instructions on how to respond emotionally to the task (for example, told to cognitively reappraise the situation). This represents an important progression in our ability to utilize fMRI to study parent-child relationships and other important social contexts using fMRI. Additional future directions of this work may examine specific populations of interest, such as adolescents who are currently depressed or at risk for mental health problems.

## Data Availability Statement

The datasets generated for this study are available on request to the corresponding author. Statistical maps can be accessed at https://identifiers.org/neurovault.collection:6150.

## Ethics Statement

The studies involving human participants were reviewed and approved by the Oklahoma State University Center for Health Sciences Institutional Review Board. Written informed consent to participate in this study was provided by participants and/or their legal guardian/next of kin.

## Author Contributions

All authors contributed to the interpretation and application of results, manuscript revision, and read and approved the submitted version. JB and MM designed and developed the concurrent fMRI scanning capabilities for conducting this study. KC, KB, DD, WS, and ASM contributed to the conception and study design including the TEAM task. KK, KB, MM, JB, and WS contributed to the data analysis. KK wrote the first draft of the manuscript. KK, KC, ER, AJM, and DD contributed to the data collection and management.

## Conflict of Interest

WS is employed by Janssen Research and Development, LLC, Johnson and Johnson, Inc. The remaining authors declare that the research was conducted in the absence of any commercial or financial relationships that could be construed as a potential conflict of interest. The handling Editor declared a shared affiliation, though no other collaboration, with one of the authors ST.

## Footnotes

1 www.pstnet.com

2 http://afni.nimh.nih.gov/afni

3 http://www.fil.ion.ucl.ac.uk/spm
